# Omalizumab treatment of moderate to severe asthma in the adolescent and pediatric population

**DOI:** 10.1186/1710-1492-10-S2-A34

**Published:** 2014-12-18

**Authors:** John O’Quinn, Stephanie Santucci, Diana Pham, Zave Chad, Ian MacLusky, Joseph Reisman, William Yang

**Affiliations:** 1 llergy and Asthma Research Centre, Ottawa, ON, Canada; 2 Department of Pediatrics, University of Ottawa, ON, Canada; 3 University of Ottawa Medical School, Ottawa, ON, Canada

## Background

In Canada and the US, omalizumab is indicated for adults and adolescents (>12 years of age) with moderate to severe persistent allergic asthma. In the EU, omalizumab has been approved for children (age 6 – 11 years) since 2009. The pediatric population within Canada and the United States has very few treatment options available for severe asthma. Current treatments options can lead to other health concerns such as adrenal insufficiency and osteoporosis. These cases demonstrate that early treatment of moderate to severe asthma with omalizumab is an effective treatment and can help to prevent or reverse damage done by long-term use of other treatment options.

## Methods

A retrospective chart review of our database was performed and patients ≤ 17 years of age receiving omalizumab treatment were evaluated. Data was collected on FEV_1_, inhaled corticosteroid (ICS) and oral corticosteroid (OCS) use.

## Results

12 patients were identified as 17 years old or younger at the start of treatment with omalizumab. After the first 6 months of treatment, all 12 patients showed an increase in FEV1 results and a decrease in ICS dose. Results also indicated a decrease in OCS use for those patients taking daily dose as well as those who required periodic bursts to control asthma exacerbations.

## Conclusion

Early treatment of moderate to severe asthma with omalizumab in adolescent/pediatric patients may improve quality of life and help prevent health concerns associated with side effects and/or long term use of ICS and OCS in growing children. Juvenile osteoporosis can be a significant problem because it occurs during the prime bone-building years and may lead to reduced peak bone mass and increased risk for osteoporosis later in life. Regular re-evaluation of the treatment regime to ensure the use of the lowest effective dose of corticosteroids and consideration of other treatments would also be beneficial.

